# Clinical Analysis of causes and preventive measures of complications after decompressive craniectomy for Craniocerebral Trauma

**DOI:** 10.12669/pjms.41.5.10382

**Published:** 2025-05

**Authors:** Zhenling Jia, Jia Li, Yuchao Shan, Dongjing Xu, Jing Li

**Affiliations:** 1 Zhenling Jia, Department of Neurosurgery Third, Baoding NO.1 Central Hospital, Baoding 071000, Hebei, China; 2 Jia Li, Department of Neurosurgery Third, Baoding NO.1 Central Hospital, Baoding 071000, Hebei, China; 3 Yuchao Shan, Department of Neurosurgery Third, Baoding NO.1 Central Hospital, Baoding 071000, Hebei, China; 4 Dongjing Xu, Ophthalmology Second Department, Baoding NO.1 Central Hospital, Baoding 071000, Hebei, China; 5 Jing Li, Ophthalmology Second Department, Baoding NO.1 Central Hospital, Baoding 071000, Hebei, China

**Keywords:** Craniocerebral Trauma, Decompressive Craniectomy, Postoperative Complications, Risk Factors

## Abstract

**Objective:**

To investigate the incidence and risk factors of postoperative complications in patients treated with decompressive craniectomy for craniocerebral injury.

**Methods:**

A retrospective analysis was conducted on the clinical data of 80 patients with craniocerebral injury who underwent decompressive craniectomy in Baoding NO.1 Central Hospital from May 2022 to January 2024, with statistics of the incidence of postoperative complications collected for the analysis of the related risk factors.

**Results:**

In this study, the incidence of postoperative complications was 37.50%, including intracranial infection (n=5; 6.25%), delayed intracranial hemorrhage (n=6; 7.50%), subdural effusion (n=15; 18.75%), cerebrospinal fluid leakage (n=3; 3.75%), and hydrocephalus (n=7; 8.75%). Additionally, the location and volume of hematoma were independent risk factors for complications after decompressive craniectomy for craniocerebral trauma(p<0.05).

**Conclusion:**

Due to the high incidence of complications after decompressive craniectomy for craniocerebral trauma, relevant measures should be taken according to the risk factors to reduce the incidence of postoperative complications, along with prompt postoperative treatment, thereby improving the efficacy of surgery.

## INTRODUCTION

As one type of traumatic disease, craniocerebral trauma(CCT) is prevalent in neurosurgery, primarily due to external force resulting in direct or indirect damage to the patient’s head.^1^,^2^ The cause of CCT-induced death is generally due to cerebral hernia secondary to intracranial hypertension, for which clinical decompressive craniectomy can rapidly reduce intracranial pressure, provide compensatory space for swollen brain tissue, and reduce compression on the brainstem vital centers.^3^

However, this therapy leads to numerous postoperative complications, such as delayed intracranial hemorrhage, external herniation of brain tissue, subdural effusion, hydrocephalus, cerebrospinal fluid incision leakage, intracranial infection, and skull defect syndrome^4^, which exhibit some negative effects on the therapeutic effect and prognosis of patients.^5^ In this study, a retrospective analysis was conducted on the clinical data of 80 CCT patients treated in Baoding NO.1 Central Hospital from May 2022 to January 2024, along with a conclusion on the incidence of postoperative complications and related risk factors in CCT patients treated with decompressive craniectomy.

## METHODS

This was a retrospective study. Eighty craniocerebral trauma patients admitted to Baoding NO.1 Central Hospital from May 2022 to January 2024 were selected as the study subjects. Among the 80 patients, there were 53 males and 27 females, aged 21 to 77 years, with a mean age of 43 years, and the primary causes of injury were traffic accidents as well as accidental falls. In the meantime, their age, gender, condition, and other clinical data were recorded completely.

### Ethics Approval:

The study was approved by the Institutional Ethics Committee of Baoding NO.1 Central Hospital (No.: [2023]085; Dated: October 23, 2023), and written informed consent was obtained from all participants.

### Inclusion criteria:

- All patients were diagnosed as CCT by head CT or MRI.
- All patients were treated with decompressive craniectomy.
- All patients and their families gave informed consent to the study content.

### Exclusion criteria:

- Patients with multiple combined organ traumas.
- A history of craniocerebral malignant tumor disease/craniocerebral operation.
- Disturbance of consciousness before craniocerebral injury.

All patients underwent decompressive craniectomy after completing further relevant examinations upon admission. All hematomas and necrotic tissues were removed intraoperatively, with a drainage tube placed under the flap after decompression, followed by skull closure through dura mater tension-reduced suture. Afterward, integrated treatments such as dehydration, anti-infection, and nutritional fluid replacement were given postoperatively. Meanwhile, all patients were followed up for six months and were divided into the observation group (those with complications) and the control group (those without complications) according to whether postoperative complications occurred.

### Outcome Indicators:

The types of complications in the observation group were statistically collected and collated according to the clinical data and follow-up data of patients, with the clinical data such as gender, age, body mass index (BMI), Glasgow Coma Scale (GCS score) at admission, and surgery-related conditions and the results of relevant examination indicators compared between the two groups, so as to investigate the related risk factors that may lead to the occurrence of postoperative complications.

### Statistical Analysis:

SPSS 22.0 statistical software was used for statistical analysis. The significance level α was set to 0.05 and the confidence interval was 95%. Measurement data were expressed as mean±standard deviation(*χ̅*±*S*), and the t-test was used for inter-group comparison. Count data were expressed as a percentage of cases[n (%)], with inter-group comparison performed using the χ^2^ test, and risk factors were analyzed using univariate and multivariate logistics regression models. P< 0.05 was considered statistically significant.

## RESULTS

Among the 80 patients in this study, thirty experienced postoperative complications, with an incidence of 37.50%, including intracranial infection (n=5; 6.25%), delayed intracranial hemorrhage (n=6; 7.50%), subdural effusion (n=15; 18.75%), cerebrospinal fluid leakage (n=3; 3.75%), and hydrocephalus (n=7; 8.75%). The results indicated that age, type of injury, timing of operation, location of hematoma, amount of hematoma, GCS score at admission, location of craniectomy flap, and degree of midline shift may be risk factors for complications after decompressive craniectomy for CCT (p< 0.05) (Table-I).

**Table-I:** Univariate Analysis of Complications after Decompressive Craniectomy for CCT [n (%)].

Factor	Observation group (n=30)	Control group (n=50)	χ^2^	P
Gender			0.032	0.859
M	18	31		
F	12	19		
Age(years)			5.895	0.015
≥50	21	21		
<50	9	29		
BMI(kg/m^2^)			0.006	0.938
≥25.00	5	8		
<25.00	25	42		
Type of injury			4.566	0.033
Open	20	21		
Closed	10	29		
Timing of operation(h)			6.519	0.011
≥12	22	22		
<12	8	28		
Location of hematoma			8.949	0.003
Epidural	4	23		
Subdural	26	27		
Volume of hematoma(ml)			8.889	0.003
≥30	25	25		
<30	5	25		
GCS score at admission(point)			6.358	0.012
≥8	23	24		
<8	7	26		
Location of craniectomy flap			4.825	0.028
Bilateral	19	19		
Unilateral	11	31		
Midline shift(cm)			4.364	0.037
≥1	21	23		
<1	9	27		

The multivariate logistics regression analysis was conducted with the presence or absence of postoperative complications as the dependent variable and the factors with statistically significant differences in the univariate analysis as independent variables (assigned values shown in Table-II). The results suggested that the location and volume of hematoma were independent risk factors for complications after decompressive craniectomy for CCT (p<0.05). Additionally, a follow-up analysis revealed that for every one mL increase in hematoma volume, the risk of complications increased about 4.7-fold (OR = 24.669). In patients with subdural hematomas, the risk of postoperative complications was 38.0% higher than epidural (OR = 14.380). Table-III.

**Table-II:** Variable Assignment Table.

Variable Label	Value Assignment	Variable Label	Value Assignment
Whether postoperative complications occurred	No = 0, Yes = 1	Location of hematoma	Epidural=0, Intradural = 1
Age(years)	<50=0, ≥50=1	Volume of hematoma(ml)	<30=0, ≥30=1
Type of injury	Closed =0, Open=1	Location of craniectomy flap	Unilateral=0, Bilateral=1
Timing of operation(h)	<12=0, ≥12=1	Midline shift(cm)	<1=0, ≥1=1
GCS score at admission	<8=0, ≥8=1		

**Table-III:** Multivariate Analysis of Complications after Decompressive Craniectomy for CCT.

Risk Factor	B	S.E.	Waldχ^2^	P	OR	95% CI
Age	0.925	0.598	2.395	0.122	2.522	0.782–8.136
Type of injury	0.201	0.693	0.084	0.772	1.222	0.314–4.749
Timing of operation	-0.116	0.925	0.016	0.900	0.890	0.145–5.453
Location of hematoma	2.666	1.044	6.519	0.011	14.380	1.858–111.294
Volume of hematoma	1.541	0.726	4.499	0.034	4.669	1.124–19.392
GCS score at admission	-0.767	1.133	0.458	0.499	0.465	0.050–4.281
Location of craniectomy flap	-0.336	0.806	0.173	0.677	0.715	0.147–3.470
Midline shift	0.087	0.869	0.010	0.920	1.091	0.199-5.986

## DISCUSSION

In this study, 30 of 80 CCT patients suffered postoperative complications, with an incidence of 37.50%, mainly including delayed intracranial hemorrhage, intracranial infection, cerebrospinal fluid leakage, subdural effusion, and hydrocephalus.^6^-^8^ Therefore, it is of great clinical significance to look for the risk factors associated with the incidence of complications and take corresponding measures to reduce the incidence of complications after decompressive craniectomy in CCT patients. Decompressive craniectomy has been developed for more than a century in the history of clinical medicine^9^, which is commonly used for CCT and various types of severe cerebrovascular diseases and can improve the prognosis and survival rate of patients.

Subdural effusion is a common complication after decompressive craniectomy, with an incidence of 26% - 60%^10^, and this study reported an incidence of 18.75%. The mechanism of subdural effusion remains unclear, which is generally believed to occur due to the following reasons: arachnoid membrane forms a unidirectional valve due to trauma or surgical tear, and cerebrospinal fluid enters the subdural space and cannot reflux, leading to subdural effusion^11^; pressure difference between the two hemispheres of the brain occurs intraoperatively, resulting in increased subdural space and ultimately effusion; atrophy of the brain tissue and drainage occurs postoperatively, leading to cerebrospinal fluid accumulation after brain tissue damage cannot be recovered.^12^ For a small volume of effusion, compression bandaging of the head or lumbar puncture or lumbar drainage can be performed to reduce the incidence of subdural effusion, while cranioplasty or effusion drainage must be performed immediately in severe cases.

The incidence of postoperative hydrocephalus in this study was 8.75%, with the main cause being the reduced cerebrospinal fluid outflow due to the destruction of intracranial pressure dynamics by craniectomy. In the meantime, a study has shown^13^ that appropriate compression bandaging of the decompression window using elastic bandages + cotton pads postoperatively can reduce the incidence of hydrocephalus. Delayed intracranial hematoma mostly occurs within 24hours postoperatively and is common in clinical practice, with an incidence of 7.50% reported in this study. The cause of bleeding is incomplete hemostasis during surgery or unstable blood pressure control,^14^ in addition to the large resected bone flap, resulting in a significant reduction of intracranial pressure, which is why meticulous intraoperative operation and attention to hemostasis are required.

Additionally, due to the incidence of 1%-5% for intracranial infection after decompressive craniectomy, the occurrence of intracranial infection may trigger meningitis and brain abscesses and affect the surgical prognosis.^15^ Therefore, the aseptic operation and minimum operation time must be strictly implemented during the surgery. Moreover, cerebrospinal fluid leakage is also a common complication after decompressive craniectomy, with an incidence of approximately 4.1%.^16^,^17^ This study reported an incidence of 3.75%, which was attributed to poor dura mater suture, high tension of postoperative encephalocele incision, too small incision suture spacing, excessive electrocoagulation of scalp hemorrhage, and ischemia around the incision due to long-term use of scalp clips.

It has been reported in some studies^18^ that the incidence of complications after decompressive craniectomy may be closely related to the age, condition, and surgery-related conditions of patients, and the univariate analysis in this study showed that age, the type of injury, timing of surgery, location of hematoma, volume of hematoma, GCS score at admission, location of craniectomy flap, and degree of midline shift may all be risk factors for complications after decompressive craniectomy, which are generally consistent with the findings of previous studies.^19^ Meanwhile, relevant studies have shown^20^ that the location of hematoma exhibits a great impact on complications, as subarachnoid edema will lead to fibrous adhesions and block villi, resulting in cerebrospinal fluid circulation disorders and ultimately hydrocephalus.

The volume of hematoma is an essential indicator to assess the severity of illness in patients with traumatic craniocerebral injury, with a greater volume of hematoma indicating a more serious condition for the patient and a higher likelihood for the incidence of postoperative complications. Moreover, this multivariate analysis demonstrated that the incidence of complications after decompressive craniectomy was closely related to the location and volume of hematoma, which was consistent with the literature.^20^

### Limitations:

However, since this study also comes with the limitations of a small number of observed cases and short follow-up time, it is necessary to expand the sample size and increase the follow-up time in the future, so as to further explore the risk factors for complications after decompressive craniectomy.

## CONCLUSIONS

Complications after decompressive craniectomy are common in CCT patients, and the location and volume of hematoma are critical risk factors. Therefore, close attention should be paid to the incidence of postoperative complications, especially for patients with subdural hemorrhage and a large hematoma volume, sufficient attention should be paid in clinical practice.

### Authors’ Contributions:

**ZJ** and **DX:** Carried out the studies, participated in collecting data, and drafted the manuscript and are responsible and accountable for the accuracy or integrity of the work.

**JL, YS** and **JL:** Literature search, Performed the statistical analysis and participated in its design. Critical Resview.

All authors read and approved the final manuscript.
